# Contactless detection of periodic leg movements during sleep: A 3D video pilot study

**DOI:** 10.1111/jsr.12986

**Published:** 2020-02-04

**Authors:** Stefan Seidel, Heinrich Garn, Markus Gall, Bernhard Kohn, Christoph Wiesmeyr, Markus Waser, Carmina Coronel, Andrijana Stefanic, Marion Böck, Markus Wimmer, Magdalena Mandl, Birgit Högl, Gerhard Klösch

**Affiliations:** ^1^ Medical University of Vienna Vienna Austria; ^2^ AIT Austrian Institute of Technology GmbH Vienna Austria; ^3^ Kepler University Hospital Linz Austria; ^4^ Medical University of Innsbruck Innsbruck Austria

**Keywords:** arousal, automatic movement detection, polysomnography, restless sleep, video

## Abstract

In clinical practice, the quality of polysomnographic recordings in children and patients with neurodegenerative diseases may be affected by sensor displacement and diminished total sleep time due to stress during the recording. In the present study, we investigated if contactless three‐dimensional (3D) detection of periodic leg movements during sleep was comparable to polysomnography. We prospectively studied a sleep laboratory cohort from two Austrian sleep laboratories. Periodic leg movements during sleep were classified according to the standards of the World Association of Sleep Medicine and served as ground truth. Leg movements including respiratory‐related events (A1) and excluding respiratory‐related events (A2 and A3) were presented as A1, A2 and A3. Three‐dimensional movement analysis was carried out using an algorithm developed by the Austrian Institute of Technology. Fifty‐two patients (22 female, mean age 52.2 ± 15.1 years) were included. Periodic leg movement during sleep indexes were significantly higher with 3D detection compared to polysomnography (33.3 [8.1–97.2] vs. 30.7 [2.9–91.9]: +9.1%, *p* = .0055/27.8 [4.5–86.2] vs. 24.2 [0.00–88.7]: +8.2%, *p* = .0154/31.8 [8.1–89.5] vs. 29.6 [2.4–91.1]: +8.9%, *p* = .0129). Contactless automatic 3D analysis has the potential to detect restlessness mirrored by periodic leg movements during sleep reliably and may especially be suited for children and the elderly.

## 1 INTRODUCTION

Periodic limb movements during sleep (PLMS) are short involuntary movements occurring repetitively mostly at about 20‐ to 40‐s intervals. Clinically, they usually consist of big toe extension and dorsiflexion of the ankle with occasional flexion at the knee and hip (Bara‐Jimenez, 2000). The prevalence of PLMS in the general population has been extensively studied in the past (Haba‐Rubio, 2018; Koo, 2015; Scofield, 2008; Szentkirályi, 2019). Scofield et al. used a PLMS index of >15/hr as a cut‐off and found a prevalence of 7.6% (Scofield, 2008). Koo et al. found that 25.6% of an elderly population‐based sample had a PLMS index between 5 and 29/hr and 17% had a PLMS index of ≥30/hr (Koo, 2015).

Periodic limb movements during sleep are linked to genetic risk variants (Moore, 2014) and objective as well as subjective sleep disturbances (Haba‐Rubio, 2018). Arousals associated with PLMS have been demonstrated to be associated with increased sympathetic activity mirrored by increased heart rate and blood pressure (Pennestri, 2013; Siddiqui, 2007).

In recent years several automatic PLMS‐detection techniques have been tested and validated successfully; for example, based on software, integrated into the polysomnography (PSG) system (Stefani, 2017), stand‐alone devices (Ferri, Fulda, et al., 2016; Ferri, Rundo, et al., 2016) or actigraphy (Gschliesser, 2009). In a previous study (Garn, 2016), we showed that automatic contactless 3D movement analysis (3D) detected all leg movements recorded during PSG that had been annotated by a human scorer. Most recently, Weinreich et al. reported a high accuracy of a non‐contact device in the detection of sleep‐disordered breathing and PLMS (Weinreich, 2018).

In this prospective pilot study, we collected polysomnographic recordings of patients with PLMS and investigated if automatic 3D analysis was able to reliably detect PLMS.

## 2 MATERIALS AND METHODS

### 2.1 Patient recruitment

Between June 2014 and October 2016 we prospectively collected polysomnographic recordings of patients presenting with various sleep complaints to the sleep laboratories of the Department of Neurology of the Medical University of Vienna and the Department of Neurology II of the Kepler Medical University of Linz. Irrespective of their clinical and sleep‐related symptoms, patients had to have at least one PLM series of at least four leg movements during PSG. Patients and their polysomnographic recordings were excluded from the final analysis if clinical data were incomplete or technical problems (artifacts or loss of sensor information during a significant portion of the night) rendered the recording not useable for data extraction.

The study was approved by the ethics committees of the Medical University of Vienna (EK‐No. 1091/2014) and the state of Upper Austria (EK‐No. 254). Written informed consent was obtained from all patients participating in the study.

### 2.2 Video PSG

All subjects underwent at least one night of 8‐hr video PSG according to the American Academy of Sleep Medicine (AASM) standards (Berri, 2018). In the case of more than one PSG recording in a patient, the second PSG was used for this study, unless technical reasons prevented this. Video PSG was recorded on Somnoscreen Plus with Domino Software (Somnomedics, Randersacker, Germany) in Linz and on MEPAL Mobil with Rembrandt Software (MAP Hirsch Medizintechnik, Villach, Austria) in Vienna. Recordings consisted of electrooculography, electroencephalography (F3, F4, C3, C4, O1, O2, M1 and M2 electrodes), cardiorespiratory signals (single channel electrocardiography), nasal air flow (thermocouple), nasal pressure cannula, thoracic and abdominal respiratory movements (piezo), transcutaneous oxygen saturation, electromyography (EMG) including at least the mental, submental and both tibialis anterior (TA) muscles, and time‐synchronized digital videography. Videotaping was performed with infrared cameras (Sony IP Camera ER521P in Linz; High Speed Dome AU‐G65 in Vienna). Leg movements were recorded using surface electrodes placed longitudinally and symmetrically around the middle of the tibialis anterior muscle, 2–3 cm apart. For scoring of EMG activity, bipolar surface EMG was recorded with the low pass filter at 100 Hz, the high pass filter at 10 Hz and a sampling rate of 500 Hz. Amplification was set at 10 μV per mm. Impedance of surface EMG electrodes had to be lower than 10 kΩ. Two experienced somnologists (SS and MB) selected the final dataset.

### 2.3 3D analysis

**Figure 1:**
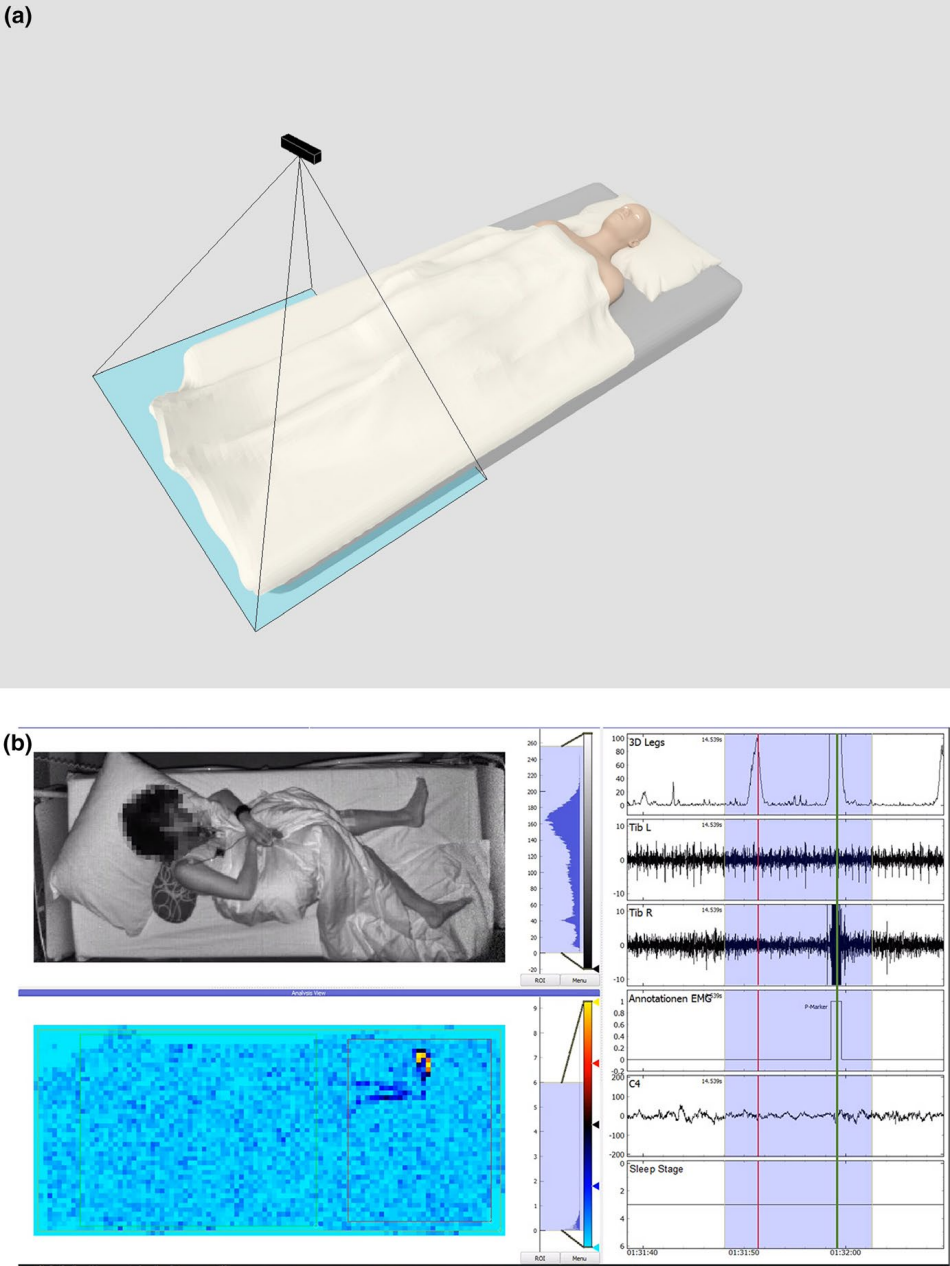
(a) Setup of the three‐dimensional (3D) sensor above the bed in the sleep laboratory. (b) Graphic example of the comparison of 3D and polysomnography (PSG) recordings. Left: images created by the 3D sensor. Top: near‐infrared video. Bottom: colour‐coded motion detection (red = motion). Right: time course of 3D and PSG signals. 3D Legs: 3D‐detected leg motion. Tib L, Tib R: electromyography (EMG) signals from left and right m. tibialis anterior. C4: central electroencephalogram (EEG) channel. Hypnogram: indicates sleep stage (rapid eye movement [REM] ‐ phase 5). Movement at 01:31:51 (red line): anteflexion of the toe, movement clearly visible in PSG video and in 3D, no detection in EMG of tibialis anterior (TA) muscles. Movement at 01:32:00 (green line): dorsiflexion of the toe, movement clearly visible in PSG video and in 3D, detection also in EMG of TA muscles. [Videos can be viewed at www.sleepmotionanalyzer.com.]

The 3D sensor was mounted on the ceiling above the bed at a distance of about 1.8 metres (Figure 1a). Three‐dimensional recordings were time‐synchronized with the PSG by feeding a time synchronization signal from the clock of the 3D system into a separate channel of the PSG headbox. We used the time‐of‐flight (TOF) sensor (Schwarte, 2001) Microsoft Kinect One (Payne, 2014) for motion detection. This sensor emits weak, amplitude‐modulated incoherent near‐infrared (IR) light at a wavelength of 860 nanometers. Its radiation intensity is far below current safety standards (BS EN 14255‐1 2005) for optical radiation. The surface of the scene reflects the light and this light is measured by a matrix of detector diodes. The electronic circuits behind each pixel provide both grey‐scale values and the time‐of‐flight of the light, which is used to compute the distance between sensor and reflecting surface. From the data of 512 × 424 pixels, our software computes a grey‐scale video and a 3D depth image for each frame at 30 frames per second. Thereby, changes in the scene can reliably be detected. Temporal changes of the distances reflect movements, which can be assigned to specific body parts. Figure 1b shows how events were presented and compared in EMG, IR‐video and 3D.

### 2.4 Computerized scoring algorithm for PLMS detection and analysis

Three‐dimensional and polysomnographic data were processed by Austrian Institute of Technology‐developed software written in Python 3.4 for detecting and localizing movements in 3D and comparison of detections in 3D and EMG. Leg movements detected by 3D were also visually inspected by co‐author MG to exclude noise‐related artefacts. Well aware of the necessity to include wakefulness and rapid eye movement (REM) sleep according to World Association of Sleep Medicine (WASM) criteria (Ferri, Fulda, et al., 2016; Ferri, Rundo, et al., 2016), we only analysed artefact‐free non‐REM sleep for this pilot study for the pragmatic reason that PLMS occur more frequently during non‐REM sleep.

“Leg movements” in the EMG signals of the PSG recordings, which were carried out according to AASM standards (Berri, 2018) (see 2.2), were visually verified by authors MB and SS and served as the ground truth for the automatic quantification of 3D leg movements. Candidate leg movements in the PSG (CLM_PSG) were selected according to WASM criteria (Ferri, Fulda, et al., 2016; Ferri, Rundo, et al., 2016), with a duration of 0.5–10 s for the unilateral and 0.5–15 s for the bilateral case. Leg movements detected in 3D with duration of 0.5–15 s were defined as CLM_3D.

According to WASM criteria (Ferri, Fulda, et al., 2016; Ferri, Rundo, et al., 2016) three different methods of scoring leg movements related to respiratory events were used and the results of the leg movement (LM) procedure are presented in the following fashion.

A1: LM including respiratory‐related events
A2: LM excluding respiratory‐related events (−2.0 to 10.25 s around the end of a respiratory event)
A3: LM excluding respiratory‐related events (−0.5 to + 0.5 s around the end of a respiratory event)

In accordance with the criteria of the WASM (Ferri, 2016), we also computed PLMS arousal indexes (PLMS_AI, median [interquartile range, IQR]). The definition of an arousal was based on the scoring rules of the AASM Manual for the Scoring of Sleep and Associated Events (Berri, 2018).

### 2.5 Statistical analysis

Normal distribution of data was tested with the Shapiro‐Wilk test with a significance level of alpha = 0.05. Accordingly, mean or median values were calculated depending on whether the samples were normally distributed (=mean) or not (=median).

Significant differences between 3D and PSG of the same measure (CLM, PLMS, PLMSI and PLMS_AI) were tested using the Wilcoxon signed‐rank test as individual distributions that were not normally distributed. Again, we used a significance level of alpha = 0.05.

Differences between 3D and PSG were calculated as follows: the numerical difference of candidate leg movements (delta CLM) was calculated by subtracting candidate leg movements detect by PSG (CLM_PSG) from candidate leg movements detect by 3D (CLM_3D).

The numerical difference of periodic leg movements (delta PLMS) was calculated by subtracting periodic leg movements detected by PSG (PLMS_PSG) from periodic leg movements detect by 3D (PLMS_3D).

The difference of the periodic leg movement index (delta PLMSI) was calculated by subtracting the PSG‐derived periodic leg movement index (PLMSI_PSG) from the 3D‐derived periodic leg movements index (PLMSI_3D).

The difference of the periodic leg movement arousal index (delta PLMS_AI) was calculated by subtracting the PSG‐derived periodic leg movement arousal index (PLMS_AI_PSG) from the 3D‐derived periodic leg movement arousal index (PLMS_AI_3D).

Correlation analyses used Spearman's rank‐order correlations coefficient to test for non‐correlation. The test provides a correlation coefficient (*r*) and a *p*‐value. We tested the correlation of resulting delta metrics: the numerical difference of candidate leg movements (delta CLM), the numerical difference of periodic leg movements (delta PLMS), the difference of the periodic leg movement index (delta PLMSI), the difference of the periodic leg movement arousal index (delta PLMS_AI) and the following PSG parameters: sleep efficiency (SE), arousal index (AI), the minimum O_2_ saturation (minSaO_2_), the mean O_2_ saturation (meanSaO_2_) and the apnea–hypopnea index (AHI).

Commonly used classification scores derived from PSG and 3D annotations measured the performance of the 3D method. PSG annotations served as the ground truth values. True positive counts (TP) were defined as events where a 3D movement overlaps with a TA EMG activity in the PSG. However, if two separated 3D movements were detected during the TA EMG activity in the PSG, only the first 3D movement was scored as TP, whereas the second was scored as a false‐positive count (FP). FP counts also included 3D movements not overlapping with TA EMG activity in the PSG. False‐negative counts (FN) presented TA EMG activity in the PSG not overlapping with any movement detected by the 3D camera/algorithm.

## 3 RESULTS

### 3.1 Patients

Complete 3D and video PSG recordings of 65 consecutive patients were collected for the study. After careful review of the PSG data, 13 (20%) patients were excluded from the study because they did not have a sufficient number of leg movements during the PSG to analyse (see Methods). Thus, 52 patients (22 female, mean age 52.2 ± 15.1 years) were included in the final analysis. Sleep‐related diagnoses comprised insomnia (*n* = 7), sleep apnea (*n* = 23), restless legs syndrome (*n* = 8), narcolepsy (*n* = 3), idiopathic hypersomnia (*n* = 1), REM sleep behaviour disorder (*n* = 1), periodic limb movement disorder (*n* = 1) and no sleep disorder according to ICSD‐III (*n* = 8). Descriptive polysomnographic data of these 52 patients are given in Table 1.

**Table 1:** Polysomnographic data of 52 patients included in the final analysis

	Hypersomnia (*n* = 3)	Narcolepsy (*n* = 4)	RLS (*n* = 7)	PLMD (*n* = 1)	Insomnia (*n* = 6)	No sleep disorder (*n* = 7)	OSA (*n* = 22)
Sex (f:m)	1:2	1:3	5:2	1:0	4:2	4:3	5:17
Age	39.7 ± 19.2	41.3 ± 14.9	55.4 ± 13.1	45	48.5 ± 21.8	50.9 ± 19.4	57.1 ± 10.0
TST	381.6 ± 15.7	330.1 ± 82.5	298.0 ± 134.3	437.0	281.6 ± 103.7	379.0 ± 78.2	329.5 ± 73.0
SE	87.7 ± 2.4	73.2 ± 16.8	77.5 ± 12.8	90.6	64.7 ± 23.8	84.3 ± 12.1	77.3 ± 18.1
SL	5.1 ± 1.5	10.5 ± 12.0	24.7 ± 33.1	6.5	93.7 ± 124.0	7.9 ± 4.2	27.9 ± 46.3
N1	15.4 ± 5.9	23.3 ± 14.8	22.9 ± 29.6	23.2	13.4 ± 7.0	15.7 ± 7.4	20.1 ± 20.1
N2	44.6 ± 9.6	30.5 ± 6.3	38.7 ± 27.0	54.6	35.6 ± 13.0	45.6 ± 6.9	38.4 ± 14.1
N3	27.0 ± 10.5	25.0 ± 23.8	27.4 ± 25.0	8.1	34.5 ± 19.9	22.6 ± 8.5	25.5 ± 17.2
REM	13.0 ± 5.3	21.2 ± 3.5	11.1 ± 8.9	14.1	16.6 ± 5.8	16.1 ± 6.2	16.0 ± 10.4
AI	20.4 ± 10.9	10.7 ± 8.1	10.2 ± 3.8	9.8	21.9 ± 12.9	8.7 ± 4.9	7.9 ± 4.9
PLMS_AI	7.1 ± 5.8	0.1 ± 0.1	0.8 ± 1.3	1.0	8.8 ± 11.6	0.3 ± 0.3	0.5 ± 0.6
PLMSI	33.4 ± 26.8	11.4 ± 12.0	29.7 ± 51.8	51.8	52.9 ± 83.7	17.7 ± 15.4	29.0 ± 29.0
AHI	7.1 ± 9.9	21.7 ± 12.6	7.2 ± 6.5	7.0	3.9 ± 6.5	9.7 ± 9.7	11.7 ± 11.1
Mean SaO_2_	95.0 ± 1.4	92.8 ± 1.0	94.9 ± 1.5	95.0	94.8 ± 1.7	93.4 ± 1.8	93.5 ± 2.9
Min SaO_2_	83.0 ± 14.9	82.5 ± 4.9	88.0 ± 5.2	91.0	86.4 ± 4.6	83.4 ± 7.6	85.6 ± 6.1

Abbreviations: AHI, apnea–hypopnea index; AI, arousal index; f, female; m, male; mean SaO_2_, mean pulsoxymetric arterial oxygen saturation (%);min SaO_2_, minimum pulsoxymetric arterial oxygen saturation (%);N1–3, % stage N1–3 in relation to TST; PLMS‐AI, PLMS arousal index; PLMSI, PLMS Index; SE, sleep efficiency (%);SL, sleep latency (min); TST, total sleep time (min); if not otherwise indicated, values represent means ± standard deviation.

### 3.2 Leg movements during sleep

A total of 14,523 min of non‐REM sleep were recorded, with a mean (±*SD*) of 279.3 ± 74.3 min per patient. In these 52 patients, a total of 4,716 min of artifact‐free non‐REM sleep were assessed, with a mean (±*SD*) of 90.7 ± 69.7 min per patient. During this time a total of 4,485 leg movements (LM) with a median (IQR) of 77.5 (32.3–134.0) LM per patient were annotated in the PSG recordings.

### 3.3 Comparison between PSG and 3D

#### 3.3.1 Candidate leg movements (CLM)

According to the WASM classification of CLM [18], the numbers of CLM detected in the PSG (CLM_PSG) were 3411/3032/3226 and the numbers of 3D‐detected CLM (CLM_3D) were 4342/3850/4122 for the whole sample. Comparison between PSG and 3D showed a statistically significant higher number of CLM_3D (increase by 27.3%, *p* < .001; 27.0%, *p* < .001; 27.8%, *p* < .001) (Table 2).

**Table 2:** Leg movements and indexes detected in the polysomnography (PSG) versus contactless 3D movement analysis (3D) for the whole sample (*n* = 52)

	WASM A1	WASM A2	WASM A3	*p*‐value
CLM_PSG (total number)	3,411	3,032	3,226	.0029/<.001/.0014
CLM_3D (total number)	4,342	3,850	4,122	<.001/<.001/<.001
CLM_PSG, median (IQR)	59.50 (24.00–102.25)	53.00 (18.25–94.50)	55.00 (22.00–97.75)	
CLM_3D, median (IQR)	61.00 (38.00–111.50)	57.00 (32.00–103.50)	60.50 (37.50–106.00)	
PLMS_PSG (total number)	2,468	2,147	2,305	<.001/<.001/<.001
PLMS_3D (total number)	2,861	2,436	2,661	<.001/<.001/<.001
PLMS_PSG, median (IQR)	38.00 (4.00–79.50)	27.50 (0.00–61.75)	35.50 (4.00–65.50)	
PLMS_3D, median (IQR)	33.00 (15.00–100.75)	25.50 (8.25–83.00)	33.50 (14.25–89.75)	
PLMSI_PSG, median (IQR)	30.74 (2.86–91.81)	24.20 (0.00–88.69)	29.65 (2.39–90.11)	.001/<.001/<.001
PLMSI_3D, median (IQR)	33.26 (8.06–97.27)	27.80 (4.48–86.17)	31.81 (8.06–89.50)	.001/<.001/<.001
PLMS_AI_PSG, median (IQR)	3.98 (0.87–14.46)	3.75 (0.00–12.31)	3.98 (0.33–13.70)	<.001/<.001/<.001
PLMS_AI_3D, median (IQR)	7.30 (2.99–20.25)	5.41 (1.52–13.73)	7.30 (2.85–17.22)	<.001/<.001/<.001

Abbreviations: CLM, candidate leg movements; IQR, interquartile range; PLMS, periodic limb movements during sleep; PLMSI, periodic limb movement during sleep index; PLMS_AI, periodic limb movement during sleep arousal index; WASM, World Association of Sleep Medicine.

In the whole sample, 91.0/90.5/90.7% of the CLM_PSG were also detected in 3D (true‐positive rate, CLM_TPR) and only 9.0/9.5/9.4% of the CLM_PSG were missed by 3D (false‐negative rate, CLM_FNR). Thus, the positive predictive value (CLM_PPV) of 3D was 68.7/68.4/67.8% for CLM_PSG.

In the individual patients, we found that the difference between CLM_PSG and CLM_3D (delta CLM) ranged between −31 and +170 with a median (IQR) of 11.5 (2.0–24.3)/8.0 (1.3–20.5)/12.0 (1.3–22.8) (Table 2).

Correlation analysis did not show any significant findings between the difference of CLM_PSG and CLM_3D (delta CLM) and the PSG parameters mentioned in 2.4.

#### 3.3.2 Periodic leg movements during sleep (PLMS)

According to the WASM classification of PLMS [18], the total count of PLMS detected in the PSG (PLM_PSG) was 2468/2147/2305, and the total count of 3D‐detected PLMS (PLMS_3D) was 2861/2436/2661 for the whole sample. Comparison between PSG and 3D showed a statistically significant higher number of PLMS_3D (increase by 15.9%, *p* = .0011; 13.5%, *p* = .0042; 15.4%, *p* = .0017) (Table 2).

In the whole sample 84.1/83.5/82.8% of the PLMS_PSG were also found in 3D (PLMS_TPR). For 15.9/16.6/17.3% of the PLMS_PSG there were no equivalents in 3D (PLMS_FNR). Finally, 71.9/72.9/71.06% of the PLMS_3D were also detected by PSG (PLMS_PPV).

In the individual patients we found that the difference between PLMS_PSG and PLMS_3D (delta PLMS) ranged between −37 and +104, with a median (IQR) of 5.0 (0.5–13.8)/3.0 (0.0–9.8)/5.0 (−1.8 to 12.5) (Table 2). Correlation analysis showed a significant correlation between the difference of PLMS_PSG and PLMS_3D (delta PLMS) and sleep efficiency (*p* = .012, *r* = .35/*p* = .001, *r* = .44/*p* = .024, *r* = .31).

The PLMS indexes calculated from the selected portions of non‐REM sleep (median (IQR)) in the PSG (PLMSI_PSG) were 30.7 (2.9–91.8)/24.2 (0.0–88.7)/29.7 (2.4–90.1) and in 3D (PLMSI_3D) 33.3 (8.1–97.3)/27.8 (4.5–86.2)/31.8 (8.1–89.5) for the whole sample. These numbers represent a statistically significant higher PLMSI in 3D as compared to PSG (9.1%, *p* = .0055; 8.2, *p* = .0154; 8.9%, *p* = .0129) (Table 2).

In the individual patients we found that differences between PLMSI_PSG and PLMSI_3D (delta PLMSI) ranged between −16.17 and +53.84, with a median (IQR) of 4.2 (0.1–8.5)/2.1 (0.0–7.4)/3.2 (−1.0–7.8). Correlation analysis showed a significant correlation between the difference of PLMSI_PSG and PLMSI_3D (delta PLMSI) and the sleep efficiency in A2 (*p* = .011, *r* = .35).

With 7.3 (3.0–20.3)/5.4 (1.5–13.7)/7.3 (2.9–17.2) the PLMSI_AI_3D were significantly higher than the PLMSI_AI_PSG 4.0 (0.9–14.5)/3.8 (0.0–12.3)/4.0 (0.3–13.7) (increase by 26.7%, *p* < .001; 25.1, *p* < .001; 29.1%, *p* < .001).

In the individual patients we found that differences between PLMS_AI_PSG and PLMS_AI_3D (delta PLMS_AI) ranged between −6.64 and +16.46, with a median (IQR) of 2.2 (0.0–4.3)/1.3 (0.0–3.0)/1.8 (0.0–3.8) (Table 2).

Correlation analysis did not show any significant correlation between the difference of PLMS_AI_PSG and PLMS_AI_3D (delta PLMS_AI) and the PSG parameters mentioned in 2.4.

#### 3.3.3 Intermovement intervals (IMI)

**Figure 2:**
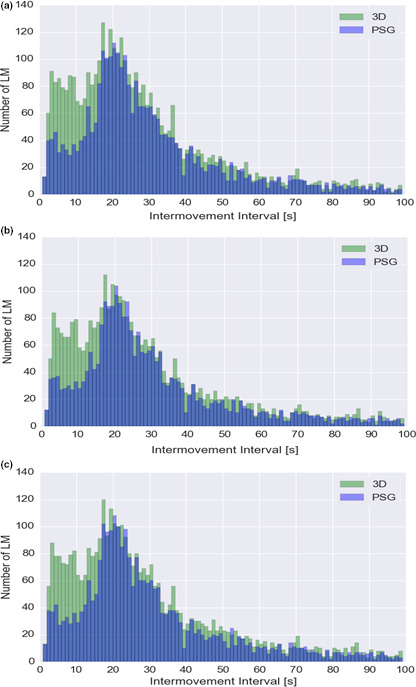
Intermovement interval (IMI) histogram of leg movements during non‐rapid eye movement (REM) sleep in all 52 patients. Panel A shows the IMI distribution for version A1, panel В shows the IMI distribution for version A2 and panel С reports the IMI distribution of version A3

Figure 2 shows the total number of CLM_PSG (blue columns) und CLM_3D (green columns) with respect to their IMI. Based on recent findings by Ferri et al., (2017, (2017b), we divided the IMI into three categories (i.e., IMI <10 s, IMI 10–90 s and IMI >90 s). According to WASM criteria (Ferri, 2016), 3D detected significantly more CLM (CLM_3D) with an IMI of <10 s than the PSG (CLM_PSG) (664 vs. 307: +116.3%, *p* < .001/576 vs. 265: +117.4%, *p* = .0014/ 619 vs. 284: +118.0%, *p* < .001).

The same applies to IMI of 10–90 s (3,080 vs. 2,554: +20.6%, *p* < .001/2,649 vs. 2,216: +19.5%, *p* < .001/2882 vs. 2,388: +20.7%, *p* < .001).

With IMI of >90 s 3D detected significantly more CLM (CLM_3D) for version A2 (573 vs. 499: +14.3%, *p* = .0445) but failed to detect significantly more CLM on the others (A1 – 546 vs. 498: +9.6%, *p* = .2242/A3 – 569 vs. 502:13.3%, *p* = .0742).

## 4 DISCUSSION

We found that automatic 3D video analysis yielded an even higher number of PLMS than PSG, especially in the short and medium IMI range. Intriguingly, and given the background of the ongoing discussion of the relationship between arousals and PLMS (Figorilli, 2017), 3D video analysis was able to detect more PLMS associated with arousals.

In clinical practice we accept PLMS associated with arousals to be clinically significant, especially if the patient complains of non‐restorative sleep and other causes of sleep disruption have been ruled out. Nevertheless, the term “clinical relevance” must be interpreted with caution. Manconi et al. (2008) showed that in patients with RLS only PLMS with an IMI between 10 and 90 s respond to dopaminergic treatment. PLMS with a short IMI of <10 s might not reflect “true” PLMS in the strictest sense, but it has also been shown that cardiac activation may be even more pronounced when induced or associated short interval leg movements (Ferri et al., 2017, 2017b).

Recently, Hooper (2018) performed a systematic video analysis of PLMS in a clinical population and proposed that the magnitude of movements should be considered as an additional factor related to the clinical significance of PLMS. Due to the exploratory nature of our study, we did not perform a systematic analysis of the composition of 3D‐detected PLMS, but the contactless approach enabled us to detect leg movements involving more proximal leg muscles than TA. Leg movements detected only in 3D but not in PSG were visually verified. Still, a certain percentage of leg movements could only be detected by the TA EMG. Applying machine learning algorithms would most likely improve the detection rate of these movements in 3D.

Although PSG undoubtedly remains the reference standard for sleep studies, our contactless 3D video analysis may be a valuable tool to study motor restlessness in special patient groups such as children and the elderly, who are both prone to anxiety and sensor displacement during a polysomnography in a restricted environment. Most recently, Del Rosso, (2018) proposed “restless sleep disorder” as a new diagnostic category, which presents with “restless sleep” or motor behaviours involving large muscle groups and consisting of frequent repositioning, moving bed sheets or even falling out of bed. Given the age range of our sample, we are not able to draw firm conclusions regarding the applicability of 3D detection of body and/or leg movements in children. Both sleep laboratories participating in the current study usually perform PSG in adults; thus we plan to cooperate with the respective paediatrics departments in future studies.

We studied a relatively small and heterogeneous sample in this pilot study. Compared to RLS cohorts (Hornyak, 2007) the PLMS arousal indexes of our patients were low, which most likely reflects the substantial proportion of sleep apnea patients in our sample, who have been reported to show lower PLMS arousal indexes (O'Brien, 2009).

A limitation of our pilot study is the fact that we only analysed non‐REM sleep. This was for the pragmatic reason that PLMS occur more frequently during non‐REM sleep and also to avoid any REM‐sleep‐associated movements and artefacts. In a further study both non‐REM and REM sleep have to be carefully analysed to comply with the full diagnostic PSG criteria of PLMS.

In conclusion, our pilot study showed that automatic 3D video analysis of PLMS works well in a sleep laboratory setting and may be a promising tool to screen patient groups such as children and the elderly for motor restlessness during sleep in a contactless fashion.

## CONFLICT OF INTEREST

Authors do not have conflicts of interest.
